# Implementation of infrared and Raman modalities for glycosaminoglycan characterization in complex systems

**DOI:** 10.1007/s10719-016-9743-6

**Published:** 2016-12-07

**Authors:** Hossam Taha Mohamed, Valérie Untereiner, Ganesh D. Sockalingum, Stéphane Brézillon

**Affiliations:** 1 Department of Zoology, Faculty of Science, Cairo University, Giza, Egypt; 2 CNRS UMR7369, Matrice Extracellulaire et Dynamique Cellulaire (MEDyC), Reims, France; 3 MéDIAN-Biophotonique et Technologies pour la Santé, UFR de Pharmacie, Reims, France; 4 Plateforme d’imagerie Cellulaire et Tissulaire (PICT), Université de Reims Champagne-Ardenne, Reims, France; 5 Laboratoire de Biochimie Médicale et Biologie Moléculaire, UFR de Médecine, Université de Reims Champagne-Ardenne, Reims, France

**Keywords:** Glycosaminoglycans, Infrared spectroscopy, Raman spectroscopy, CHO-WT, CHO-745, Chondrocytes, Conditioned media, Data analysis

## Abstract

Glycosaminoglycans (GAGs) are natural, linear and negatively charged heteropolysaccharides which are incident in every mammalian tissue. They consist of repeating disaccharide units, which are composed of either sulfated or non-sulfated monosaccharides. Depending on tissue types, GAGs exhibit structural heterogeneity such as the position and degree of sulfation or within their disaccharide units composition being heparin, heparan sulfate, chondroitine sulfate, dermatan sulfate, keratan sulfate, and hyaluronic acid. They are covalently linked to a core protein (proteoglycans) or as free chains (hyaluronan). GAGs affect cell properties and functions either by direct interaction with cell receptors or by sequestration of growth factors. These evidences of divert biological roles of GAGs make their characterization at cell and tissue levels of importance. Thus, non-invasive techniques are interesting to investigate, to qualitatively and quantitatively characterize GAGs *in vitro* in order to use them as diagnostic biomarkers and/or as therapeutic targets in several human diseases including cancer. Infrared and Raman microspectroscopies and imaging are sensitive enough to differentiate and classify GAG types and subtypes in spite of their close molecular structures. Spectroscopic markers characteristic of reference GAG molecules were identified. Beyond these investigations of the standard GAG spectral signature, infrared and Raman spectral signatures of GAG were searched in complex biological systems like cells. The aim of the present review is to describe the implementation of these complementary vibrational spectroscopy techniques, and to discuss their potentials, advantages and disadvantages for GAG analysis. In addition, this review presents new data as we show for the first time GAG infrared and Raman spectral signatures from conditioned media and live cells, respectively.

## Introduction

### Pathophysiological aspects of glycosaminoglycans

Glycosaminoglycans (GAGs) and proteoglycans (PGs) are major constituents of the extracellular matrix (ECM). PGs are present at the cell surface, in the ECM, in intracellular granules and in basement membranes [1]. GAGs are unbranched and negatively charged heteropolysaccharides composed of repeating disaccharide units of alternating uronic acids and N-acetylated hexosamine [2]. Most of GAGs are covalently attached to core proteins to form PGs [3, 4]. Numbers of attached GAG side chains vary from only one chain as decorin, a Small Leucine Rich Proteoglycan (SLRP), to more than 100 chains as aggrecan [3]. According to the types of monosaccharide in the repeating unit and their sulfation, there are different major categories of GAGs: hyaluronan (HA), dermatan sulfate (DS) and chondroitin sulfate (CS), heparin (HEP), heparan sulfate (HS) and keratan sulfate (KS) [3, 5]. KS is the only GAG that loses uronic acid and contains galactose in its disaccharide units [3]. HA is a non-sulfated GAG devoid of a covalently bound core protein. HA is synthesized at the extracellular surface of the plasma membrane and plays an important role in tissue homeostasis and cancer progression [6]. All sulfated GAGs are synthesized at the Golgi apparatus and modified by O-sulfotransferases [7]. The ionic properties due to the presence of carboxylate and sulfate groups make GAG molecules able to attract water [3]. GAGs diversity is due to the modifications in sulfation positions and epimerization of the glucuronic acids to iduronic acids [3]. The structure and abundance of GAG chains vary according to the tissue. For example, lumican displays four potential sites for the substitution by N-linked KS or oligosaccharides situated at positions 87, 126, 159 and 251 of the human core protein. In the cornea, lumican is a KSPG characterized by a smear on immunoblots, with a molecular mass ranging approximately from 70 to 300 kDa. In contrast, in skin, lumican is present in the dermis in the form of a glycoprotein (57 kDa) [8]. Proteoglycans enrolled several biological functions. They modulate cell growth-factor activation, regulate collagen fibrillogenesis, affect tumor cell growth and invasion, and influence corneal transparency. As a KSPG, lumican regulates corneal transparency [9] and stimulates keratocytes migration [10]. In contrast, as a glycoprotein, it inhibits melanoma cell migration [8]. The first known biological function of GAGs is the clinical anticoagulant role of HEP. Numerous genetic studies on GAG biosynthetic enzymes revealed direct evidences of involvement of GAGs in cell growth including transforming growth factor (TGF), and fibroblast growth factor (FGF) signalling pathways [11].

Mutations in the genes encoding sulfotransferases and glycosyltransferases enzymes caused a number of genetic disorders. Sulfotransferases are responsible for the synthesis of sulfated GAGs side chains of proteoglycans. Recently, studies revealed that mutations in genes encoding CS and DS biosynthetic enzymes cause several disorders of connective tissues [12]. In some corneal diseases, sulfated GAGs were considered to be pathogenic factors [13]. GAGs and proteoglycans play important roles in cancer progression, where changes in their expression and enzymes involved in their biosynthesis and/or degradation contribute to the cancer progression [2]. In breast cancer, overexpression of hyaluronan synthase 2 increases ErbB2-dependent signalling leading to disease progression [14], while suppression of hyaluronan synthase 2 leads to inhibition of tumorigenesis and progression of breast cancer [15]. In addition, CSPGs were shown to activate the extracellular signal–regulated kinase and focal adhesion kinase in melanoma [16]. Decorin was demonstrated to regulate epidermal growth factor receptor signalling, thus controlling proliferation in melanoma [6]. Any changes in the expression of GAGs reduce cell adhesion and increase cancer cell invasion [2]. Syndecans, acting in harmony with integrins and hyaluronan signalling through CD44 was shown to increase cancer cell motility [17]. GAGs play a major role in metastasis and angiogenesis. HA expression affects the metastatic potential of mouse mammary carcinoma cells [18]. Degradation of cell-surface HS chains and matrix HSPGs by heparanase increase invasion and metastasis [19]. Syndecan-1 regulates the adhesion of cancer cells to lymphatic vessel endothelium [20]. Inhibition of perlecan expression decreases the growth of colon carcinoma cells and tumor angiogenesis [21]. CS has an antiangiogenic effect by inhibiting the migration of transendothelial monocytes [6]. Moreover, decorin was shown to be angiostatic due to a high-affinity binding to the VEGFR-2 [22]. Decorin is an antagonistic ligand for VEGFR-2 and a 12 amino acid peptide within the leucine-rich-repeat 5 domain of decorin protein was responsible for the most avid VEGFR-2 binding, antagonizing VEGF actions [22]. In stem cells, GAGs and PGs are considered as specific markers of progenitor cells [23]. In addition, they play major supportive roles in developmental signalling and provide a sanctuary for preservation of cell “stemness” as demonstrated for CSPG in neural stem cells [24] and HSPG and CSPG in hematopoietic precursor cells [25]. These evidences of divert biological roles of GAGs make their characterization at cell and tissue levels of importance. Thus, non-invasive techniques are interesting to investigate, to qualitatively and quantitatively characterize GAGs *in vitro* in order to use them as diagnostic biomarkers and/or as therapeutic targets in several human diseases including cancer.

### Bioanalytical methods for glycosaminoglycan characterization

GAGs display an important heterogeneity in their molecular structure including within their uronic acid, hexosamine, and the position of the sulfate groups. Detection and identification of GAGs structure are crucial to understand their biological role. Extraction of GAGs is mainly performed by enzymatic depolymerisation with exogenous proteinase or sodium hydroxide [26]. Physicochemical analysis of GAGs involved ion pairing chromatography and Mass Spectrometry (MS) applied after preincubation of the samples with specific enzymes. The chemical analysis of such preparations is a tedious task involving enzymatic depolymerisation of GAG chains with specific bacterial enzymes followed by disaccharide analysis with Gel Permeation Chromatography (GPC) [27], High-Performance-Liquid Chromatography (HPLC), Ultra-Performance Liquid Chromatography (UPLC) [28], Nuclear Magnetic Resonance (NMR) spectroscopy, Mass Spectrometry (MS) [29], Capillary Electrophoresis (CE) [30] and Fluorophore Assisted Carbohydrate Electrophoresis (FACE) [31, 32].

Liquid chromatography-mass spectrometry (LC-MS) and MS, have become widely used in analysis of GAGs without interference by impurities in biological samples [33, 34]. Reverse-phase ion-pair (RPIP)-HPLC depends on volatile lipophilic ion-pairing reagents in the mobile phase to favour analyte attachment on a hydrophobic C18 stationary phase and evaporation in electrospraying, leading to make these ion-pairing reagents compatible with electrospray ionization (ESI)-MS [35, 36]. RPIP-HPLC-MS is applicable for several analytes, ranging from unsulfated to highly sulfated GAGs [28, 37]. RPIP-UPLC-MS provides even higher resolution, sensitivity, efficiency and peak capacity, at high pressures (up to 108 Pa) with columns packed with 1.7 μm particles [38]. There are several factors affecting RPIP-HPLC-MS or RPIP-UPLC-MS separation of GAGs, such as the concentration of ion pairing reagent, counter-ion and pH. Moreover, the routine structural analysis of GAGs in a cell, tissue or biological fluid sample requires multiple selective polysaccharide lyase treatments, multiple disaccharide isolation steps, multiple HPLC or UPLC columns with different mobile phases and different ESI -MS detection methods [37].

MS is another powerful technique for structural analysis of GAGs. Soft ionization techniques, including ESI and matrix-assisted laser desorption/ionization (MALDI) are more dedicated to the analysis of neutral oligosaccharides and their attached peptide and lipid conjugates [29, 39]. MALDI time-of-flight (TOF) MS [40, 41] and ESI-MS [42] are useful approaches for the structural analysis of GAG-derived oligosaccharides. Both ESI-MS and MALDI-TOF-MS are convenient for structural analysis of large polar macromolecules. NMR spectroscopy allows determining the saccharide composition, ring conformation, glycosidic linkage, and sulfation modality of GAGs [43, 44]. Regrettably, this technique is very limited due to the requirement of relatively large amounts (milligrams) of pure GAG sample.

CE is one of the most sensitive techniques for GAGs analysis due to its resolving power, separation efficiency, short time analysis and straightforward operation [45]. Moreover, CE has higher compatibility with several detection methods, such as ultraviolet (UV) spectroscopy, MS [46], NMR spectroscopy and Laser Induced Fluorescence Detection (LIF) [47, 48]. Currently, determination of GAG disaccharides using CE-LIF requires separate retrievals from multiple lyase-digestion steps, and separated analysis of HS/HP and CS/DS in two chromatographic runs, leading to a complicated and more time-consuming technique. However, the results from different analyses showed inaccuracy to the calculated ratio of HS/HP and CS/DS GAGs obtained from one sample. Chang and collaborators used a simplified procedure to do GAG-derived disaccharides analysis from biological source using a single CE-LIF experiment [49].

FACE is a reliable and rapid technique to analyse unsaturated disaccharides on the base of electrophoresis after derivatization with a fluorophore, such as AMAC. FACE presents the advantages of the fluorescence of AMAC for high sensitivities and the possibility to analyze large number of samples with one electrophoretic apparatus at the same time [50]. FACE is able to determine disaccharides from a very small quantity of sample (0.05 ng). Karousou and his colleagues demonstrated that FACE is an accurate and highly selective for CS in biological samples and established FACE technique can be easily used for the contemporaneous separation of CS/DS and hyaluronan [51]. Thus, FACE analysis is a powerful tool to characterize low quantities of different sulfated CS disaccharides.

To better understand, the roles of GAGs in physiology and pathophysiology, it is important to be able to determine the structure of GAGs from a small quantity of tissue, but even better at the cell scale according to the cell phenotype. Spectroscopic markers characteristic of reference GAG molecules have been identified previously based on their molecular structures by Raman and infrared (IR) microspectroscopies [52, 53]. These techniques are sensitive enough to descriminate different cell types according to their capacity to synthetise GAGs [54]. Both IR and Raman microspectroscopies are highly sensitive to the structure, composition, and environment of the molecules constituting the studied specimen. Moreover, these biophotonic methods are rapid, non-destructive and do not require external markers. In terms of structural and compositional analysis, IR and Raman spectroscopies are able to give a complete “molecular fingerprint” for GAGs in the studied sample.

### Principles of vibrational spectroscopy

Infrared (IR) and Raman spectroscopies are vibrational spectroscopic techniques based on light -matter interactions. They allow to probe the chemical content of all forms of matter *via* transitions in their vibrational energy states. The information obtained in the form of a spectral signature characteristic of the state, molecular structure and composition, and environment of the sample. These techniques are currently used to characterize simple molecules, complex biological macromolecules (lipids, proteins, nucleic acids, polysaccharides), and even highly complex systems like cells and tissues [55]. In infrared spectroscopy, the sample interacts with an incident light from a polychromatic source comprising a set of wavelengths that span over the mid-infrared range (2.5–25 μm). The mid- infrared source carries the energy that exactly corresponds to the energy differences between the ground state vibrational energy levels of the molecule. As a consequence, a molecule excited by a mid-infrared source may absorb this energy and in this case a specific bond will start vibrating. This is the case when a molecular vibration is infrared-active, *i.e.*, it undergoes a change in its dipole moment during this interaction. Infrared spectroscopy analyses the intensity difference between the incident light and light transmitted or reflected by the sample. The infrared spectrum therefore displays the absorption intensity at each frequency. This spectrum can be interpreted qualitatively *via* the position of the bands, which is directly related to the frequency of vibration and therefore to the chemical information, and quantitatively *via* its intensity, which can be associated to its concentration. IR spectroscopy measures the absolute frequencies of vibration.

**Fig. 1:**
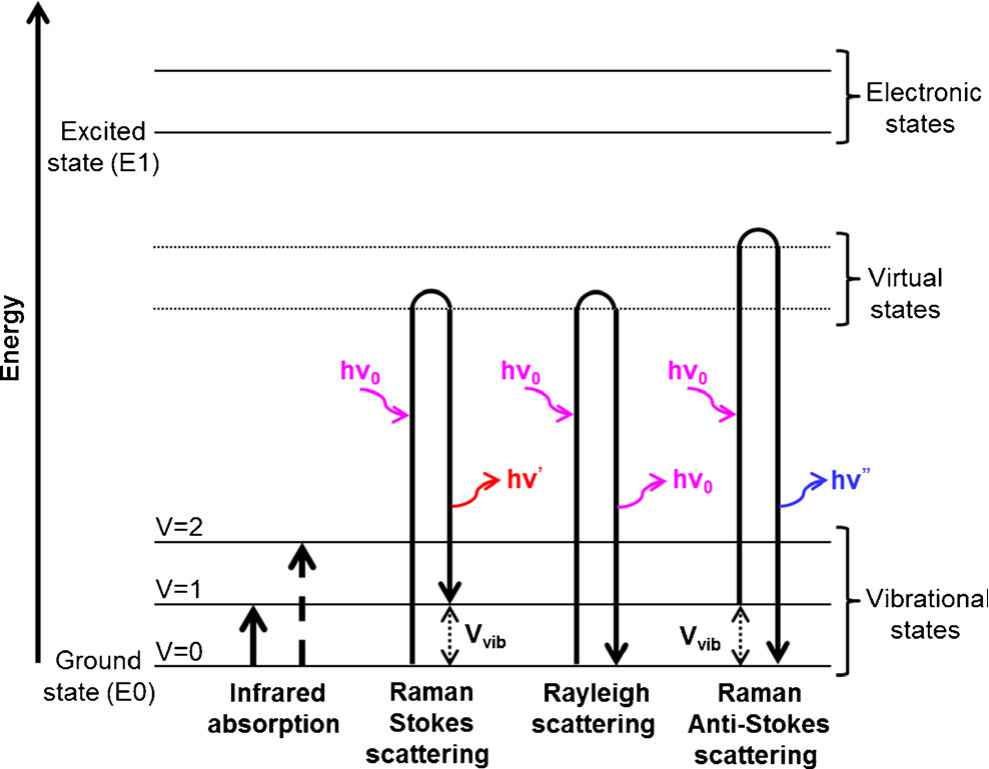
Energy diagram showing transitions involved during infrared absorption and Raman scattering processes. Rayleigh (ordinary) scattering is when the photon is absorbed to a higher virtual state and is instantly scattered (emitted) elastically back to the initial state, without any change in its frequency. The photons emitted by Raman Stokes scattering usually have a lower frequency (*red shifted*) than that of the photons absorbed and these photons are inelastically scattered, transferring some of their energy to the molecule. The reverse is also possible; the photons emitted have a higher frequency than the photons absorbed (*blue shifted*). This is called Raman Anti-Stokes, but it is less likely at room temperature as the majority of electrons are in the ground state. Both Raman Stokes and anti-Stokes give the vibrational information as the Raman shift, which is the difference between the two vibrational energy states. The difference between the ground state and the final state gives the vibrational energy (ν_vib_) of the system and corresponds to energy states measured in IR spectroscopy. The vibrational energy ν_vib_ is related to a specific molecular vibration and to the structure and the chemical composition of the molecules of the samples. *Double and dashed arrows* represent Raman shift and overtone respectively

Raman spectroscopy is a scattering process that is based on the interaction of the sample with a monochromatic source, generally a laser. During this interaction, the molecule gains energy and reaches a “polarized” or “virtual” state, which is not energetically stable and consequently the molecule comes down to the initial energy level by emission of photons. For a large majority of cases, the molecule will come back to its initial energy level, *i.e.*, the lowest vibrational energy level of the ground state. This gives rise to elastically scattered photons with the same frequency as that of the incident laser. This event is the most probable and therefore predominates and is termed as “Rayleigh scattering.” Since one gets back the same frequency as the incident excitation, Rayleigh light does not carry any chemical information. A second possibility is that the scattered light is inelastic in nature, *i.e.*, the transition to the ground state gives rise to scattered photons that have either gained energy (anti-Stokes Raman) or given away some energy (Stokes Raman). In both cases, the difference between the initial and final states gives the energy of the vibrating bond and also corresponds to the difference in energy levels measured in IR spectroscopy (see Fig. 1). However, it must be noted that, while in IR spectroscopy a measurement of the absolute frequency is made, in Raman spectroscopy the measured frequency is relative since a difference is calculated with respect to the excitation frequency. This frequency difference is also called “Raman shift.” The spectral information obtained with the anti-Stokes and Stokes scattering is identical, but since in the former the initial state of the molecules is not the lowest energy level, the anti-Stokes is less intense because the number of events involved is less. Therefore, in spontaneous Raman measurements, spectrometers are generally designed to capture mainly the Raman Stokes signal, while the Rayleigh and anti-Stokes scattering are rejected. The Raman spectrum therefore displays the Stokes scattering intensity with respect to the Raman shift. In the same way as with IR, the position of the bands is related to the chemical nature of the sample. Its intensity can be used in a semi-quantitative manner.

On the other hand, a molecular vibration is said to be Raman-active if it undergoes a change in its polarizability during the interaction. Although the origins of the physical phenomena are not the same (absorption for IR and scattering for Raman), both techniques inform on the chemical nature of the sample. They are also complementary because certain vibrational modes that are active in IR may not be active in Raman and *vice-versa*. So, combining both techniques can give the full vibrational signature. These signatures consist of several vibrational modes of molecular linkages in a molecular group: symmetrical or antisymmetrical stretching, scissoring, rocking, wagging, and twisting. Depending on the applications, lasers for Raman spectroscopy can range from the UV to the near-infrared. UV and visible lasers produce photons of higher frequency and can therefore cause sample heating. Also, the “polarized” state can coincide with the first excited electronic level giving rise to fluorescence emission, which will mask the Raman signal. Sample degradation and fluorescence can be circumvented by increasing the excitation wavelength to the near-infrared (less energetic photons).

### State-of-the-art of application of vibrational spectroscopy to the study of glycosaminoglycans

The first IR spectra of GAGs were published more than 50 years ago [56]. Later studies analysed the differences of the spectra in solid-state and in aqueous solutions [57], or with different hydration rates [58–63] or from different species (CS from different sources of cartilage, such as crocodile, shark, chicken, ray) [64]. Several bands were identified by spectral comparison [65] especially those associated with sulfate vibrations [66–70] and different conformations of iduronate [71–74]. Prepared GAGs [70] or GAGs present in natural, PG forms [75] were characterized. The evolution of molecular changes with aging was also studied [76, 77]. More recently, the association of chemometrics with sensitive spectroscopic microtechniques showed a good classification of some types of GAGs [75–78]. These studies concerned only a particular type of GAGs or digested molecules. The first IR spectral signatures of GAG in complex biological systems like cells were also recently demonstrated [54]. However, IR microspectroscopy is limited in terms of spatial resolution and also requires sample drying. In order to study live cells, Raman microspectroscopy (RMS) is potentially interesting to perform studies in physiological conditions. Raman spectra of GAGs were published 38 years ago [66]. It is highly sensitive to the structure, composition, and environment of the molecules constituting the studied specimen [79].

The association of spectroscopy with powerful data analytical methods gives more insight into the interpretation of the spectral information and molecular-level phenomena. When coupled with a microscope, vibrational microspectroscopies become highly sensitive methods capable of probing at the micron level, thus necessitating only small amounts of sample. Raman and Raman optical activity of GAGs was described [80]. RMS was used to study spectral signatures of various mixtures of GAGs with collagens [81]. Raman spectral imaging of single living cancer cells was previously described [82–85]. Moreover, vibrational microspectroscopy was shown to be both qualitative and semi-quantitative. Surface-enhanced Raman scattering (SERS) was used to map glycan expression for the identification of cancerous cells [86]. Raman SERS was reported to be highly sensitive to detect HEP [87].

The aim of the present review is to describe the implementation of these complementary vibrational spectroscopies (VS) techniques and to discuss their potentials, advantages and disadvantages for GAG analysis. In addition, this review presents new data as we show for the first time GAG infrared and Raman spectral signatures from conditioned media and live cells (CHO-745, CHO-WT and chondrocytes), respectively.

## Vibrational spectroscopy: sample preparation for Raman and infrared analysis

CHO-WT cell line and its mutant counterpart, CHO-745, lacking xylosyltransferase associated with low GAG synthesis and primary culture from human chondrocytes expressing high levels of GAGs were used in the study.

### Extraction of total GAGs and quantification of sulfated GAGs

**Fig. 2:**
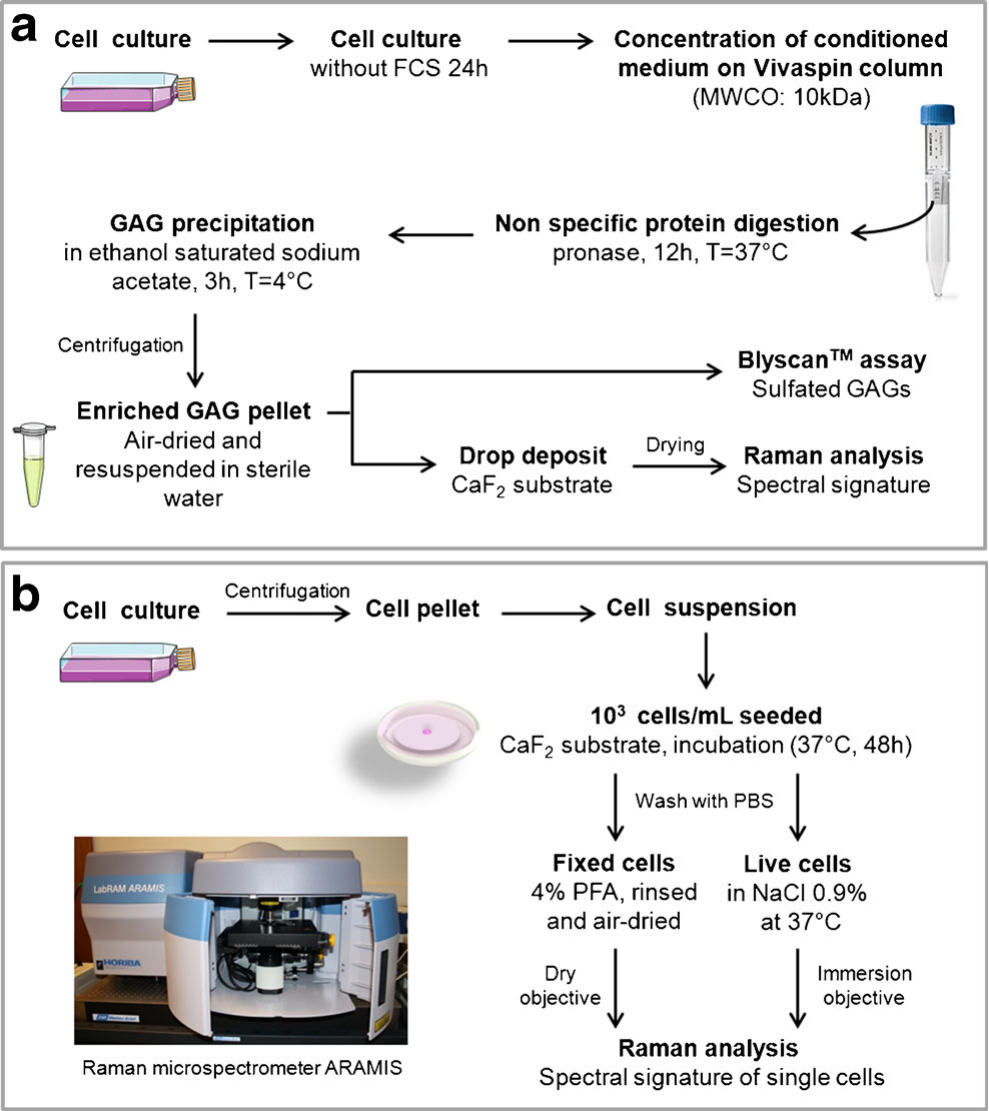
Workflow from conditioned media to biochemical analysis and Raman spectral analysis (**a**) and from cell suspension to Raman spectral analysis of fixed and live cells (**b**)

The workflow for the extraction and quantification of sulfated GAGs is given in Fig. 2a . Cells underwent starvation in growth media without serum for 24 h at 37 °C with 5 % CO_2_. Then, conditioned media (CM) were collected and were centrifuged to discard the cell debris. The collected CM was concentrated using Vivaspin™ column (10,000 Da MWCO) (GE Healthcare Bio-Sciences AB, Sweden). CM was incubated overnight with proteinase at 37 °C for a non-specific protein digestion. Proteinase digestion was stopped by addition of NaCl (50nM) and incubation at 100 °C for 1 min. After cooling down, the samples were centrifuged to pellet the digested proteins. GAGs were precipitated from the supernatant by addition of ethanol saturated sodium acetate and incubation at 4 °C for 3 h. Purified GAGs were centrifuged and air-dried. Dried GAG pellets were resuspended in sterile distilled water. A Blyscan™ assay (Biocolor, Westbury, NY, USA) was performed to determine the concentration of sulfated GAGs. The absorbance was measured at 656 nm from 3 replicates for each sample.

### Sample preparation for Raman microspectroscopy

The workflow for micro-Raman analysis of extracted GAGs (Fig. 2a) involves the following steps. Three drops of 2 μL of resuspended extracted GAGs were deposited on CaF_2_ substrate and air-dried. The measurements were performed with a Raman microspectrometer (Horiba JobinYvon, Villeneuve d’Ascq, France) equipped with an Olympus microscope (model: BX40) and a dry objective (X100, NA = 0.9). The source was a 660 nm diode laser (Fig. 2b). The schematic for the micro-Raman analysis of single fixed and live cells is illustrated in Fig. 2b. At 80 % of confluence, cells were detached and cell pellets were collected after centrifugation. Cells were resuspended in complete cell culture medium, seeded on CaF_2_ substrate (10^4^ cells/mL) and incubated for 2 days at 37 °C with 5 % CO_2_. For fixed cells analysis, cells were fixed with 4 % paraformaldehyde (PFA) and air-dried. For live cells, the substrate was washed then immersed in 0.9 % NaCl solution. The Raman analysis was done by using a water-immersion objective lens.

### Sample preparation for infrared analysis

**Fig. 3:**
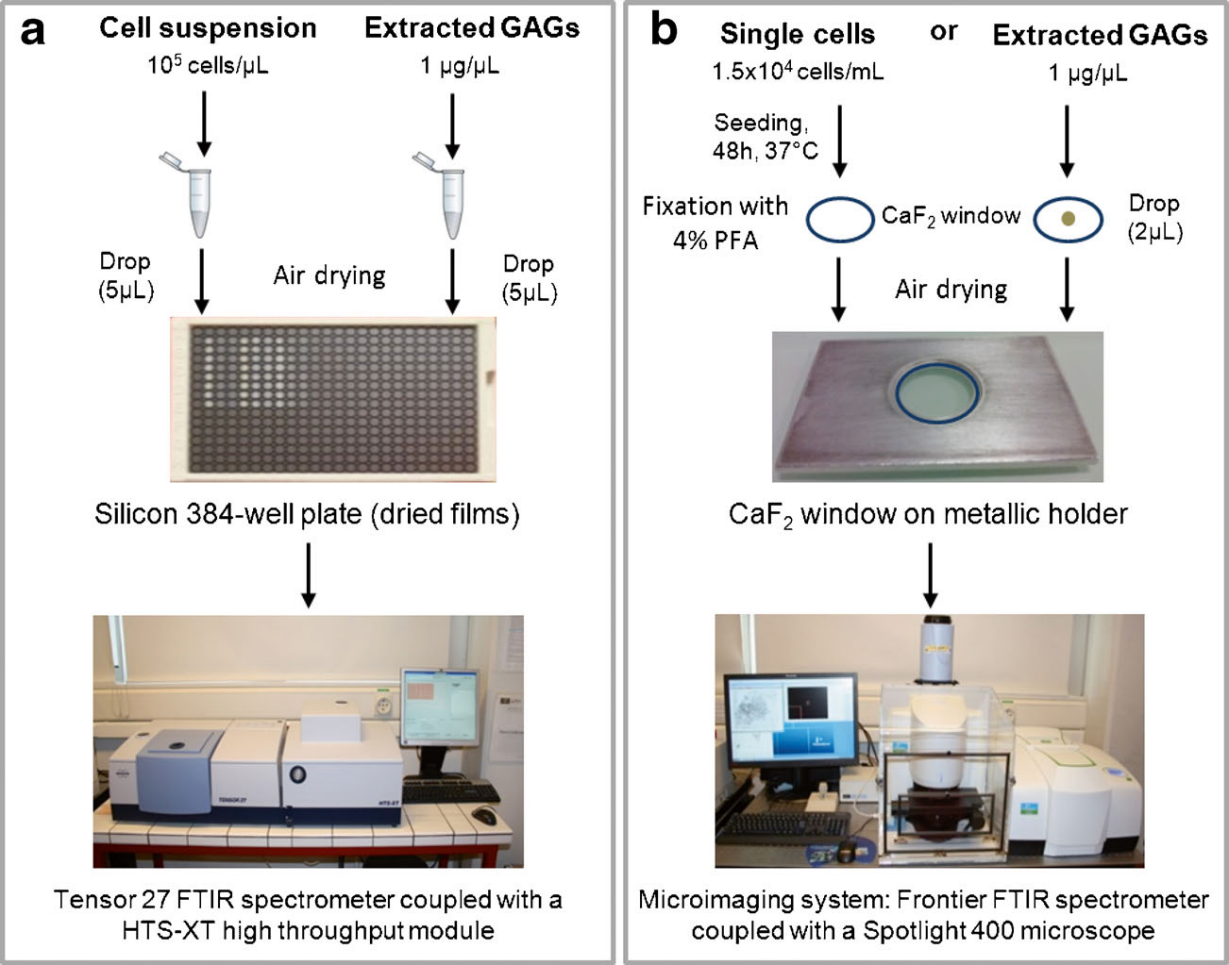
Workflow from the cell suspension or extracted GAGs to high-throughput FTIR spectral analysis (**a**) and from single cells or extracted GAGs to FTIR microimaging system (**b**)

The workflow for cell and extracted GAGs preparation for FTIR analysis is given in Fig. 3. Five μL of cell suspension (10^5^ cells/μL) or 5 μL of re-suspended GAGs extract (1 μg/μL) were analysed with a high-throughput screening HTS-XT extension coupled to a Tensor 27 FTIR spectrometer (Bruker Optics GmbH, Etlingen, Germany). The samples were deposited in triplicate onto a high-throughput 384-well silicon plate and air-dried (Fig. 3a). Single cells and extracted GAGs (Fig. 3b) were analysed with an FTIR microimaging system Spotlight 400 (Perkin Elmer, Courtaboeuf, France). Cells (1.5 × 10^4^ cells/mL) were seeded on a CaF_2_ substrate for 48 h at 37 °C. Then, they were fixed with 4 % PFA and air-dried. For extracted GAGs, three drops of 2 μL were deposited on a CaF_2_ window and air-dried.

## Vibrational spectroscopy and data acquisition

### Infrared spectroscopy data acquisition

The IR acquisitions of the samples were performed in transmission mode, in the spectral range 4000–400 cm^−1^, at a spectral resolution of 4 cm^−1^, with 64 scans and 128 scans for extracted GAGs and cells, respectively. Before each sample measurement, the silicon plate background was recorded and automatically removed from the sample signal. One spectrum was obtained from each well. Acquisitions were performed with the OPUS software (Version 6.0, Bruker Optics, Germany). For FTIR imaging, the analysis was acquired in transmission mode at a spatial resolution of 6.25 μm/pixel for cells and at 25 μm/pixel for GAG extracts. The spectral range 4000–800 cm^−1^ was used at a spectral resolution of 4 cm^−1^. For fixed cells and extracted GAGs, 128 scans and 16 scans were used, respectively. Spectral images of fixed cells were taken from the whole single cell and spectral images of GAG samples were taken across the diameter of each dried drop. The substrate background is automatically subtracted from the signal. The images of single cells or GAG drops were averaged and the obtained spectra were pre-processed.

### Raman microspectroscopy data acquisition

All Raman measurements were carried out with a 660 nm laser excitation. The spectral resolution was 4 cm^−1^ giving by a grating of 950 lines/mm. For extracted GAGs, an acquisition of 30 spectra per dried drop was performed in the point mode over the 600–1750 cm^−1^ spectral range with an accumulation time of 60s. For fixed and live cells, the acquisitions were performed in the point mode (spatial resolution of ~1 μm) from cell cytoplasms over the 600–1750 cm^−1^ spectral range and an acquisition time of 45 seconds/point. For extracted GAGs and fixed cells, a X100 dry objective (NA = 0.9) was used while a X100 immersion objective (NA = 0.9) was employed for live cells measurements.

## Spectral data pre-processing and analysis

**Fig. 4:**
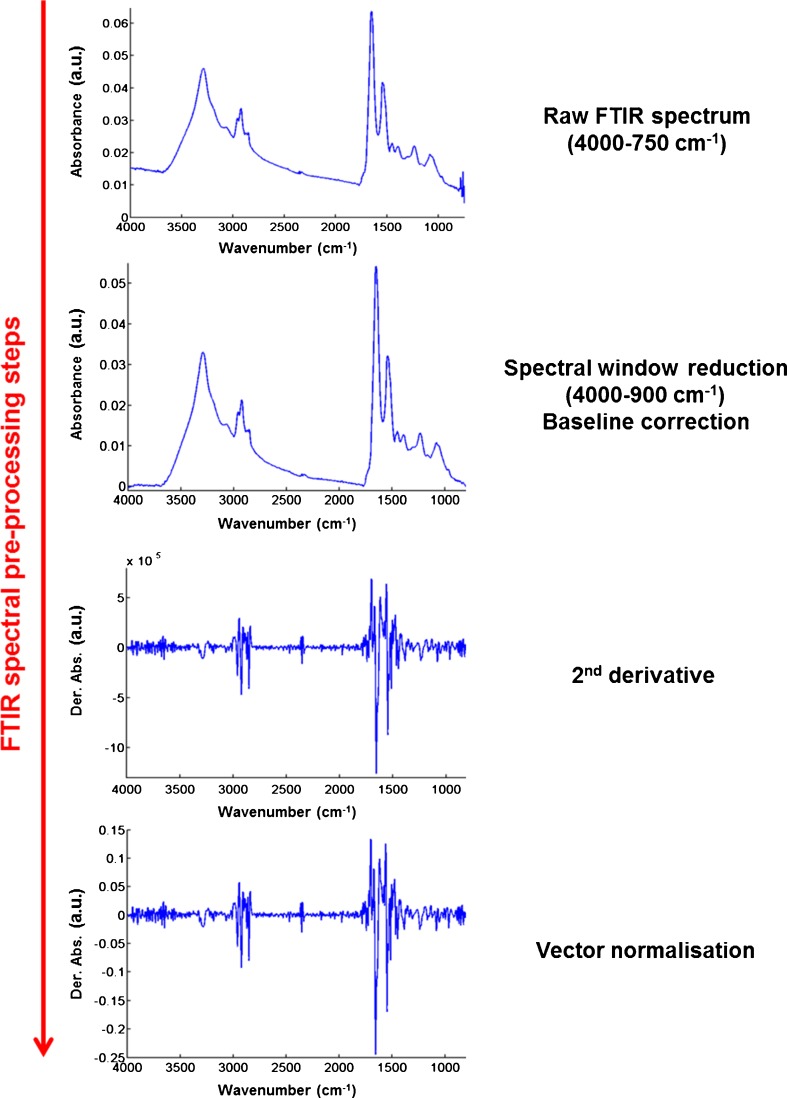
Steps for pre-processing of an FTIR spectrum. Example is given for a single fixed CHO-WT cell

For FTIR and Raman analysis, the pre-processing was specific for each technique. Figure 4 shows the workflow for pre-processing of a typical FTIR spectrum. An example is shown for a single fixed CHO-WT cell. This was carried out with the OPUS software (Version 6.0, Bruker Optics, Germany). Raw spectra, initially recorded between 4000 and 750 cm^−1^ were reduced to the 4000–800 cm^−1^ spectral window to remove the noisy end of the spectrum. Spectra were then baseline corrected (elastic function, 9 points), their second derivative computed to increase spectral differences, and vector normalised.

**Fig. 5:**
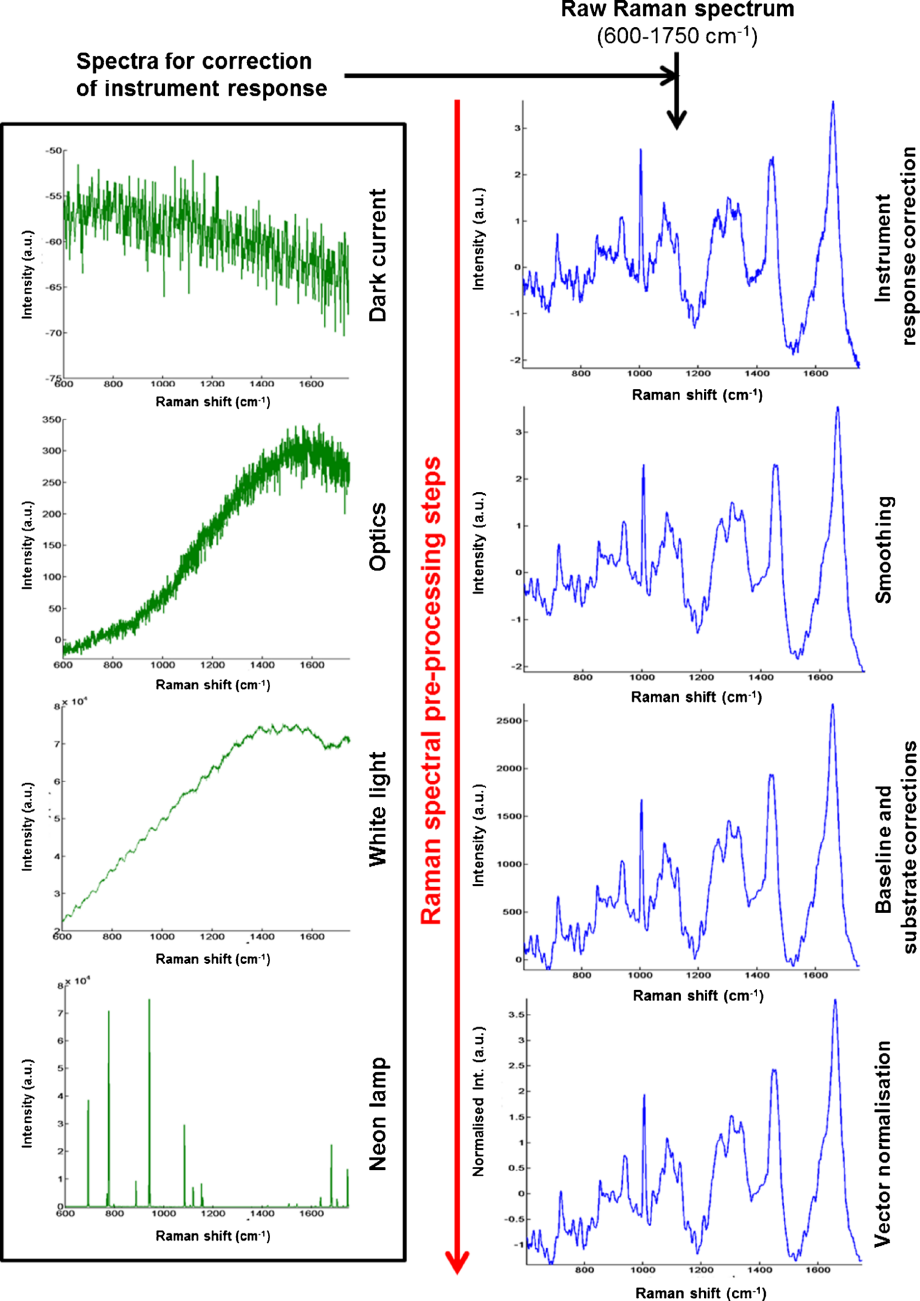
Steps for pre-processing of a Raman spectrum. Example is given for a single live CHO-WT cell

Figure 5 shows the workflow for pre-processing of a typical Raman spectrum. An example is shown for a single live CHO-WT cell. This was carried out with a home-made software using the Matlab environment (The MathWorks, Inc., Natick, USA). Raw spectra were recorded between 600 and 1750 cm^−1^. Before each Raman experiment, the following spectra were recorded to correct for instrument and substrate contributions: dark current (measurement without laser and objective) and optics (with laser and objective) to estimate the noise, white light (halogen lamp) for intensity calibration, neon lamp for frequency calibration, and finally the CaF_2_ window to remove substrate contribution. Each raw Raman spectrum undergoes these steps before smoothing with a Savistsky-Golay function (polynom order 3 and window 9) to reduce noise, baseline and substrate subtraction, and vector normalisation on the whole spectral range.

After this first step, data from these two techniques were processed with exploratory techniques like hierarchical cluster analysis (HCA) and principal component analysis (PCA). These clustering methods based on distance calculation and variance analysis, respectively, allowed to view, in an unsupervised way, how spectra group together. HCA computes the Euclidian distance between the spectra using the Ward’s algorithm. This classification allows the combination of spectra with a minimum Euclidian distance in the same cluster. The distance between each group gives an estimate of the spectral difference between spectral clusters. The result of clustering is represented in a tree called dendrogram. PCA is a statistical analysis based on the orthogonal transformation to convert a group of observations of possible correlated variables into a group of linearly uncorrelated principal components (PCs). PCA is an exploratory method and can be performed by singular value decomposition of a data matrix after mean centering. Results of a PCA are presented as a plot of PC scores or PC loadings.

## Infrared and Raman microspectroscopies: rapid and non-invasive screening techniques distinguishing cell types exhibiting low to high levels of GAG synthesis

In terms of structural and compositional analysis, VS is able to give a complete “molecular fingerprint” of the studied sample, depending on their molecular structure. We previously showed that VS such as IR and Raman microspectroscopies are promising techniques to characterize, differentiate, and classify GAGs types and subtypes despite their close molecular structures [52]. Some characteristic spectral regions and bands were identified and used in combination with chemometric methods such as HCA and PCA, to characterize standard GAGs by VS [52, 53]. These approaches could be extremely useful for performing quality tests on GAG samples. Moreover, VS was shown to be both qualitative and quantitative.

Brézillon and collaborators [54] applied IR microspectroscopy and imaging to identify in a first step the spectroscopic signatures of GAG standards, GAG-Collagen mixtures, and in a second step cells expressing different levels of GAGs, both in suspensions and in isolated single cells. Cartilage chondrocytes are the cells with the highest capacity for GAGs synthesis. The CHO-745 cell line, lacking xylosyl transferase, is the only known mutant deficient in the synthesis of CS and HS which results in a decreased amount of GAGs in these cells, compared to the wild type CHO (CHO-WT). We first reported on IR spectroscopy of major types of GAGs, *i.e.*, HA, HEP, HS, C4S, C6S, DS and GAG-collagen mixtures. We next investigated cellular IR spectroscopy of CHO-745, CHO-WT, chondrocytes [54] and SK-MEL-28, dermal fibroblast (unpublished data) in suspension and performed IR imaging of single cells. Spectral data were analysed by HCA chemometric method in order to characterize these different cell types exhibiting from low to high levels of GAG synthesis. We identified the infrared spectral signatures of standard GAGs and differentiated cell types based on their capacity of GAG synthesis [54].

**Fig. 6:**
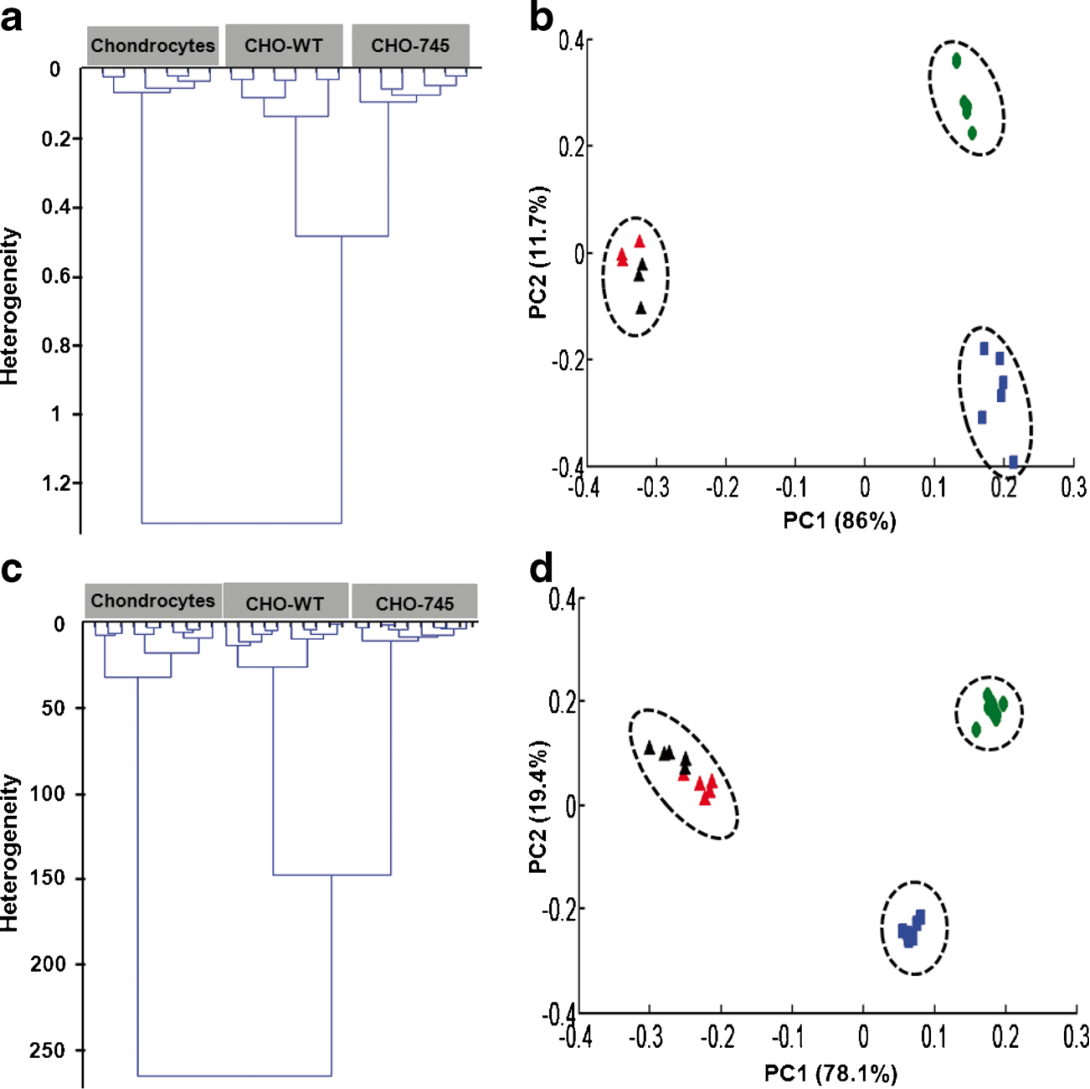
Hierarchical cluster and principal component analyses from FTIR spectra of CM extracted GAGs and single cells. HCA (**a**) and PCA (**b**) analyses from FTIR spectra of CM extracted GAGs (n = 6) obtained from CHO-745, CHO-WT and chondrocytes. HCA (**c**) and PCA (**d**) analyses from FTIR spectra of fixed CHO-745, CHO-WT and chondrocytes (n = 10). *Red and black triangles* correspond to two different individuals. HCA and PCA analyses were calculated on the second derivative of the mean spectra of each cell type in the spectral range 1350–900 cm^−1^. All replicate spectra of the same sample are well clustered together

In addition to the previous study performed on fixed cells [54], we show in the present review in Fig. 6, CHO-745, CHO-WT, and chondrocytes CM characterized by their ability of synthesizing GAGs from very low to high levels. Normalized FTIR spectra of these cell types were obtained from GAGs extracted from CM (Fig. 6a and b) and single cell analyses (Fig. 6c and d), respectively. Visually, the spectral profiles (not shown) of chondrocytes, CHO-WT and CHO-745 were similar in the 1800–900 cm^−1^ spectral range, with, however, some minor modifications for chondrocytes in the 1350–900 cm^−1^ spectral range (GAGs absorption region). The extracted GAGs from CM and the fixed cells FTIR spectra were analysed by the HCA analysis (Fig. 6a and c). This method was performed on all second derivative spectra for each single cells and for CM in the 1350–900 cm^−1^ spectral range. The spectra of cells and CM were grouped in the same cluster and exhibited a low degree of heterogeneity, indicating a good reproducibility of the measurements and, therefore, a low intra-group variability (Fig. 6a and c). The inter-group variability was sufficiently high to distinguish the three cell types (Fig. 6a and c). The dendrogram also revealed the inter-group variability enabling to differentiate between chondrocytes and both CHO-WT and CHO-745 at both single cells and CM levels. The clear-cut classification using the second derivative spectra in the 1350–900 cm^−1^ spectral range indicated that chondrocytes differed from CHO-WT and CHO-745 by their spectral differences in the GAGs absorption region. Moreover, these FTIR spectra were analysed by PCA (Fig. 6b and d). PCA analysis for single cells and CM showed low intra-group variability in both CHO cell types while higher variability was observed in chondrocytes. This result might be explained at least in part by inter-individual differences from one patient to another one and the degree of cell differentiation. In addition, the inter-group variability was sufficiently high to distinguish the three cell types. The PCA score plot of the three cell types showed that the first two PCs exhibited the highest explained variance. For CM, the PC1 carried 87 % of the total variance and allowed clear separation of CHO-745 and CHO-WT from chondrocytes. These results also demonstrated that PC2, representing 12 % of the total variance, discriminated efficiently CHO-745 and CHO-WT. For single cells, PC1 representing 89 % of the total variance, allowed clear separation of CHO-745 and CHO-WT from chondrocytes. The PC2 representing 9 % of the total variance could separate CHO-745 and CHO-WT. Therefore, the capacity of GAG synthesis of different cell types can be clearly delineated using HCA and PCA analyses. Therefore, this methodology was demonstrated to be a useful approach for screening and identifying cells that exhibit the capacity for GAG synthesis. Furthermore, the method is rapid, non-destructive, non-contact, and label-free. Taking into account the importance of GAGs in the biomedical field, it will be challenging, to transpose this methodology to tissue analysis in order to identify GAG molecules in normal tissues and during pathophysiological conditions.

Since IR microspectroscopy is limited in spatial resolution and requires sample drying, RMS is potentially interesting to perform studies in physiological conditions. The application of Raman spectroscopy and microscopy within biology is rapidly increasing because it can provide chemical and compositional information. Moreover, it does not suffer from water interference. In addition, it does not require extensive sample preparation. Biochemical and structural information can be obtained without labelling [88].

**Fig. 7:**
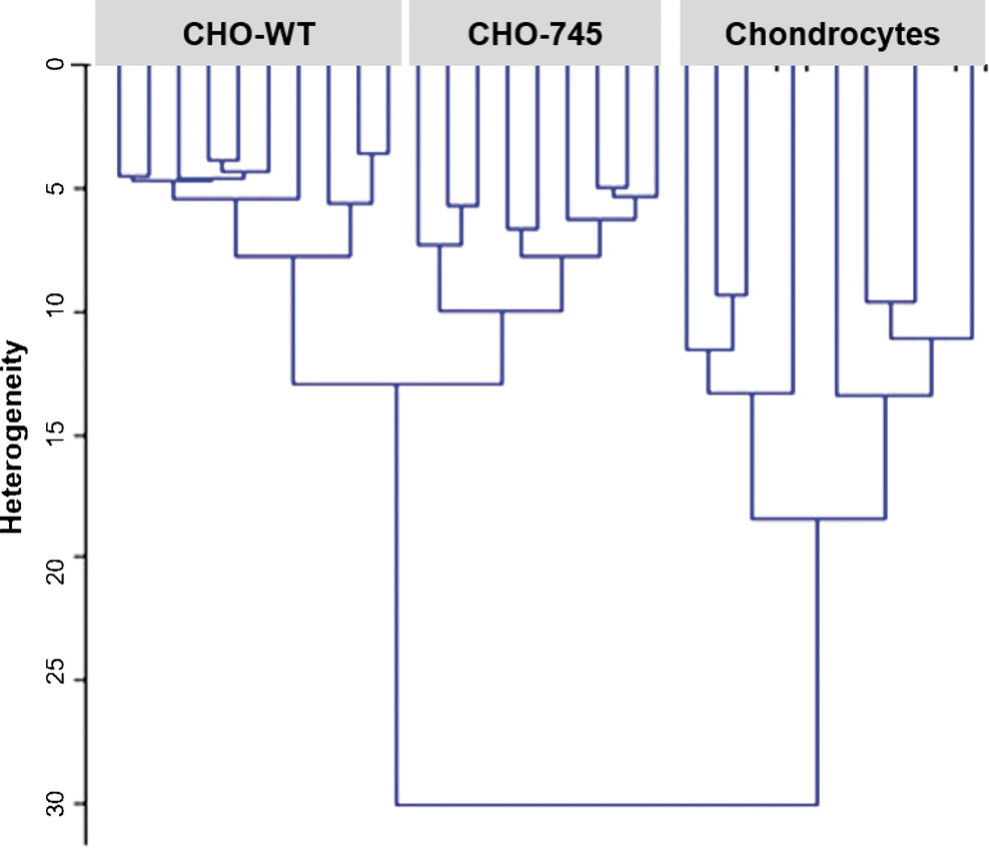
Hierarchical cluster analysis of live cells Raman spectra. HCA was calculated on the second derivative of the mean spectra of each cell type in the spectral range 600–1750 cm^−1^. All replicate spectra of the same sample are well clustered together

Raman spectroscopy is an analytical tool for cancer research [89]. Real-time *in vivo* cancer diagnosis using Raman spectroscopy is currently developing [87, 90]. It is now applied to the analysis of biofluids [91]. The combination of Raman spectroscopy, which measures molecular vibrations, and Raman optical activity, which measures the small difference in Raman Scattering from chiral molecules using circularly polarized light, can provide information about the structure and conformational dynamics of biological molecules such as GAGs [80]. In the present review, we analysed for the first time normalized Raman spectra from single live cells of CHO-745, CHO-WT, and chondrocytes. The Raman analysis was performed at 1750–600 cm^−1^ spectral range. In Fig. 7, HCA analysis showed a low degree of heterogeneity, indicating a good reproducibility of the measurements and, therefore, a low intra-group variability. The inter-group variability was good enough to distinguish the three cell types (Fig. 7). The dendrogram clearly differentiated between chondrocytes and both CHO-WT and CHO-745. PCA (not shown) demonstrated the prominence of the information related to GAGs.

FTIR and Raman spectroscopies are emerging analytical techniques that are complementary, straight-forward, and easy-to-use. They allow molecular characterization of GAG either as standards or in complex biological systems such as cells. FTIR can be used either in a high-throughput approach for analyzing CM and fixed cell populations or in the imaging mode, where isolated fixed single cells can be investigated. In this case, the spectral information obtained is a snapshot of the differentiation or proliferation state of the cell. Thus, it is possible to analyze intra- and peri-cellular GAGs at a given time. The FTIR spectral data allow to screen and to discriminate cells according to their capacity of GAG synthesis. FTIR is sensitive to the degree of sulfation of GAGs. IR microspectroscopy of live cells is feasible but remains challenging.

Complementary to FTIR, RMS also allows to analyze CM and cells. However, RMS offers a better spatial resolution, which renders measurements in subcellular compartments. In addition, water being a weak scatter, investigation of live cells in physiological conditions is feasible. Thus, GAGs can be analyzed in a native conformation unaltered by chemical fixation. In a complementary way, RMS is more sensitive to glycosidic bonds. In this study, we have used the 660 nm excitation but other lasers from the visible to the near-IR can be adapted to analyze intra- and peri-cellular GAGs.

## Conclusions and outlook

As succinctly reviewed here, high-throughput FTIR and Raman spectroscopies have evolved to a point at which cells with different capacities of GAG synthesis can be discriminated. Thus, it is possible, *via* the use of two different chemometric clustering approaches, to distinguish between the CHO-WT cell line and its mutant counterpart, CHO-745, lacking xylosyltransferase associated with low GAG synthesis. The inter-group differences were more marked when the latter two cell lines were compared to the primary culture from human chondrocytes expressing high levels of GAGs. Therefore, VS could be developed for cell screening purposes distinguishing cell types exhibiting from low to high levels of GAG synthesis and further for identifying GAG molecules in tissues. Taking into account the importance of GAGs in the biomedical field, it will be challenging and further work is needed to transpose the methodology to tissue analysis in order to identify GAG molecules in normal tissues and during pathophysiological conditions. In a mid-term perspective, *in vivo* RMS could be developed to search for GAG spectral signatures in peritumoral margins of certain tumors as a marker of tumor progression.

## Abbreviations

CE: Capillary electrophoresis
CM: Conditioned medium
CS: Chondroitin sulfate
DS: Dermatan sulfate
ESI: Electrospray-ionization
ECM: Extracellular matrix
EGF: Epidermal growth factor
FACE: Fluorophore assisted carbohydrate electrophoresis
LC-MS: Liquid chromatography-mass spectrometry
FGF: Fibroblast growth factor
FTIR: Fourier transform infrared
GAG: Glycosaminoglycan
GPC: Gel permeation chromatography
HA: Hyaluronic acid
HCA: Hierarchical cluster analysis
HEP: Heparin
HS: Heparan sulfate
HPLC: High-performance-liquid chromatography
IR: Infrared
KS: Keratan sulfate
LIF: Laser induced fluorescence
MALDI: Matrix-assisted laser desorption/ionization
MS: Mass spectrometry
MWCO: Molecular weight cut off
NMR: Nuclear magnetic resonance
PCA: Principal component analysis
PFA: Paraformaldehyde
PG: Proteoglycan
RMS: Raman microspectroscopy
RPIP: Reverse-phase ion-pair
SLRP: Small leucine rich proteoglycan
TGF: Tumor growth factor
UPLC: Ultra-performance liquid chromatography
VEGF: Vascular endothelial growth factor
VS: Vibrational spectroscopy
